# Managing unplanned radiotherapy interruptions in Italy: results from an AIRO survey

**DOI:** 10.1007/s12094-025-04211-6

**Published:** 2026-02-01

**Authors:** Francesco Deodato, Alba Fiorentino, Donato Pezzulla, Gabriella Macchia, Stefania Manfrida, Nicola Dinapoli, Mattia Falchetto Osti, Giuseppe Sanguineti, Alessio Giuseppe Morganti, Elvio Grazioso Russi

**Affiliations:** 1 Radiation Oncology Unit, Responsible Research Hospital, Campobasso, Italy; 2 Istituto di Radiologia, Università Cattolica del Sacro Cuore di Roma, Rome, Italy; 3 Radiation Oncology Department, General Regional Hospital F. Miulli, Acquaviva Delle Fonti, Italy; 4 Radiation Oncology Unit Gemelli ART, Fondazione Policlinico Universitario A. Gemelli IRCCS, Rome, Italy; 5 Radiation Oncology Unit, Sapienza Università di Roma, AOU Sant’Andrea, Rome, Italy; 6 Department of Radiation Oncology, IRCCS Regina Elena National Cancer Institute, Rome, Italy; 7 Radiation Oncology Unit, Department of Experimental, Diagnostic and Speciality Medicine – DIMES, S. Orsola-Malpighi Hospital, University of Bologna, Bologna, Italy; 8 Teaching Hospital “S. Croce e Carle”, Cuneo, Italy

**Keywords:** Radiation therapy interruptions, Treatment compensation strategies, LINAC breakdowns, National survey

## Abstract

**Background:**

Radiation oncology (RO) is essential in cancer treatment. Unplanned interruptions reduce tumor control, yet no validated management guidelines exist. The Italian Association of Radiation and Clinical Oncology (AIRO) conducted a national survey to assess the prevalence, causes, and management strategies for RT interruptions across Italy.

**Methods:**

This cross-sectional survey was conducted between April and June 2022. A 34-question survey was emailed to directors of all Italian ROT centers, covering: (1) demographic and institutional characteristics; (2) radiobiological knowledge of ROT interruptions; (3) clinical management and compensation strategies.

**Results:**

A total of 104 centers responded. Respondents had a median age of 57 years (range 34–74), and most worked in General Hospitals (64%). Centers had a median of 2 LINACs (range 1–6), with 16% operating only one LINAC. 96% of radiation oncologists (Ros) considered ROT interruptions a critical issue, particularly in curative (51%) and adjuvant (29%) settings. 42% defined an interruption of > 5 days as critical, and 63% believed treatment phase did not influence impact. 29% of ROs followed formal guidelines [e.g., Royal College of Radiographers (RCR)]. The main causes of interruptions were LINAC breakdowns (22%), toxicity (22%), and patient compliance issues (22%). 24% of ROs followed codified procedures for managing interruptions; 84% regularly monitored treatment breaks. Dose recovery strategies: 28% always compensated, 59% occasionally compensated, primarily by increasing total dose (48%) or working on Saturdays (20%).

**Conclusion:**

This study reveals variability in ROT interruption management, stressing the need for AIRO guidelines, collaboration, and modern radiobiological integration.

**Supplementary Information:**

The online version contains supplementary material available at 10.1007/s12094-025-04211-6.

## Introduction

Radiation oncology (RO) is a critical component of cancer treatment and is prescribed to more than 50% of all oncology patients [1]. Unplanned interruptions during the RO treatment (ROT) course extend the overall treatment time, leading to a reduction in tumor control probability due to tumor repopulation. This phenomenon has been observed across various tumor types, particularly in squamous cell carcinomas [2–4]. Each day of interruption has been associated with a progressive decline in expected survival outcomes [4–19]. However, no validated measures currently exist to predict the clinical impact of RT interruptions.

When ROT breaks are unavoidable, compensatory treatment may be advisable. Although several compensation strategies exist, their feasibility is often constrained by practical considerations, such as machine availability, the possibility of extending operational hours or working on weekends, and patient-specific clinical factors that may influence the selection of the most appropriate compensation method [20].

The issue of ROT interruptions and recovery strategies became particularly pressing during the COVID-19 pandemic, prompting the publication of several single-institution experiences in the literature [13, 21]. However, most of these studies reported monocentric approaches, limiting a comprehensive understanding of the current landscape of unplanned ROT interruptions, their causes, and their management strategies.

Given the absence of national guidelines for managing ROT interruptions, the Italian Association of Radiation and Clinical Oncology (AIRO) initiated a national survey to investigate this issue. The survey aimed to identify the primary causes of ROT interruptions and to assess the strategies employed at a national level to mitigate or eliminate their clinical impact.

## Materials and methods

This project was conducted under the leadership of the Lazio-Abruzzo-Molise Regional Group of AIRO. An external panel of radiation oncologists (ROs) with specific expertise in the management of treatment interruptions reviewed the survey, providing feedback and recommendations.

The survey consisted of 34 questions divided into 3 sections, designed to assess participants’ knowledge and clinical practices regarding ROT interruptions. The first section (questions 1–9) collected demographic and professional information for stratification purposes (Table 1). The second section (questions 10–15) evaluated the ROs’ radiobiological knowledge on the topic (Table 2). The third and final section (questions 16–34) investigated the protocols and procedures adopted by each center in cases of ROT interruptions (Table 3). A complete list of the survey questions is provided in Supplementary Table 1.

**Table 1:** Respondents’ demographic and professional information

Category	*N*	%
Sex		
Male	64	62
Female	40	38
Median age (range)	57 years (34–74)	
Type of hospital		
General hospital	67	64
Private contracted/private hospital	23	22
University hospital	14	14
Geographical distribution of centers		
Located in Northern Italy	41	39
Located in Central Italy	27	26
Located in Southern Italy/Islands	36	35
Number of LINACs per center		
1 LINAC	17	16
2 LINACs	53	51
More than 2 LINACs	34	33
Year of LINAC installation^a^		
Between 2000 and 2010	59	31
Between 2011 and 2015	46	24
Between 2016 and 2020	57	30
From 2021 onward	30	15
Availability of dosimetric “Twin” LINACs		
Present	47	45
Not available	57	55
Management of concomitant therapy and supportive care (multiple responses possible; total responses: 139)^b^		
Regular hospital beds designated for inpatient care	9	7
Beds available in the outpatient/day hospital unit	20	14
Direct management of supportive care and systemic outpatient therapies by the RO department	25	18
Supportive therapy and systemic therapies managed by other specialists in separate departments	71	51
Other	14	10
Operating schedule of the RO center		
5 days/week	76	73
6 days/week	5	5
5 days/week with potential recovery sessions on Saturdays	23	22
Typical start day for RO treatments		
Monday	3	3
Any day of the week	88	85
Other	13	12

*LINAC* linear accelerator, *RO* radiation oncology

^a^Year of installation was assessed based on when each LINAC was introduced at the center

^b^Percentages were calculated based on the total number of responses (*N* = 139) for this question

**Table 2:** Radiation oncologists’ radiobiological knowledge

Category	*N*	%
Consideration of treatment interruptions as a management issue		
Treatment interruptions considered an issue	100	96
Treatment interruptions not considered an issue	4	4
Situations where interruptions are considered critical (multiple responses possible; total responses: 194)^a^		
Radical treatment	99	51
Adjuvant treatment	56	29
Palliative treatment	39	20
Tumor types considered most affected by treatment interruptions (multiple responses possible; total responses: 431)^a^		
Breast cancer	29	7
Prostate cancer	21	5
Cervical cancer	86	20
Rectal cancer	64	15
Head and neck (HeN) cancers	97	23
Brain cancers	47	11
Lung cancers	78	18
Other	9	1
Number of interruption days considered to have a negative impact on treatment outcomes		
2 days	5	5
3 days	21	20
5 days	34	33
More than 5 days	44	42
Phase of treatment where interruptions are considered to have a negative impact		
At any phase of treatment	65	63
At the start of treatment	4	4
In the middle of treatment	22	21
At the end of treatment	13	12
Use of bibliographical references or guidelines for managing treatment interruptions and dose compensation		
Guidelines or references followed	30	29
No guidelines or references followed	74	71

*HeN* head and neck

^a^Percentages were calculated based on the total number of responses for each multiple-choice question (*N* = 194 for situations; *N* = 431 for tumor types)

**Table 3:** Procedures followed in case of treatment interruption

Category	*N*	%
Availability of written procedures for managing ROT interruptions		
Written procedures available	25	24
No written procedures available	79	76
Monitoring of treatment interruptions		
Patients’ treatment interruptions are regularly monitored	87	84
No monitoring of treatment interruptions	17	16
Most frequent causes of treatment discontinuation (multiple responses possible; total responses: 387)^a^		
Equipment breakdown	85	22
Logistical problems	53	14
Low patient compliance	84	22
Holidays	52	13
Toxicity	84	22
Other	29	7
Management of ROT interruptions during the COVID-19 pandemic (before vaccine availability)		
Treatment halted for all COVID-19-positive patients until negative test result	50	48
Treatment stopped only for symptomatic COVID-19-positive patients	29	28
Treatment continued for all COVID-19-positive patients	15	14
Other	10	10
Management of ROT interruptions during the COVID-19 pandemic (after vaccine availability)		
Treatment halted for all COVID-19-positive patients until negative test result	29	28
Treatment stopped only for symptomatic COVID-19-positive patients	45	43
Treatment continued for all COVID-19-positive patients	21	20
Other	9	9
Formalized agreements for patient transfer in case of LINAC downtime (centers with only one LINAC, *N* = 17)		
Agreements in place with other centers	4	24
No agreements in place	13	76
Management of patients during LINAC downtime		
Patients transferred to another LINAC	85	82
Patients not transferred to another LINAC	19	18
Selection criteria for moving patients to another LINAC (*N* = 85)		
All patients transferred	21	25
Only specific cases transferred	64	75
Use of specific criteria for selecting patients to move to another LINAC (*N* = 85)		
Formalized procedures followed	18	21
Non-formalized procedures followed	54	64
No specific procedures followed	13	15
Criteria used for selecting patients to move to another LINAC (multiple responses possible; total responses: 193)^a^		
Primary tumor	50	26
Exclusive/adjuvant treatment	48	25
Symptomatic versus asymptomatic patients	31	16
Concomitant systemic treatment	54	28
Other	10	5
Recalculation of treatment plan before moving a patient to a second LINAC (*N* = 85)		
Treatment plan recalculated using TPS	61	72
No recalculation performed	24	28
Pre-calculation of treatment plan for two different LINACs before treatment start (*N* = 85)		
Pre-calculation performed	7	8
No pre-calculation performed	78	92
Recovery of missed radiation dose due to treatment interruption		
Always performed	29	28
Performed only in specific cases	61	59
Never performed	2	2
Only missed sessions are recovered by extending treatment duration	12	11
Methods used for dose recovery (multiple responses possible; total responses: 142)^a^		
Treatment extended to following Saturdays	28	20
Two daily applications on one or more days	15	10
Accelerated regimen (hyperfractionation or not) applied in remaining treatment time to maintain overall duration	13	9
Total dose increased to recover lost dose, without changing fractionation	68	48
Total dose increased with modified fractionation (e.g., hyperfractionation)	18	13
Patient information in case of different daily fractionation for dose recovery (*N* = 46)		
Patient informed, no new consent required (modifications included in original informed consent)	35	76
Patient informed, new informed consent required	9	20
Patient not informed	2	4
Personnel responsible for managing equipment failures		
Clinical engineering department	36	35
Technical service department	4	4
Coordinator of medical radiology health technicians	48	46
Medical physics department	14	13
Other	2	2
Availability of preventive maintenance contracts for LINACs		
Preventive maintenance contract in place	104	100
No preventive maintenance contract	0	0
Entity responsible for performing preventive maintenance		
LINAC manufacturer	101	97
Technical service department	1	1
External service provider	2	2
Scheduling of preventive maintenance for LINACs		
Conducted on any day of the week	103	98
Conducted only on Saturdays	2	2

*LINAC* linear accelerator, *TPS* treatment planning system, *ROT* radiation oncology treatment

^a^Percentages were calculated based on the total number of responses for each multiple-choice question

The survey was distributed via email by the AIRO group to the director of each Italian RO center, inviting voluntary participation between April and June 2022. The online, cross-sectional survey was conducted using SurveyMonkey (www.surveymonkey.com; accessed in April 2022), which automatically recorded responses. Professional data were securely stored within the SurveyMonkey platform and protected against unauthorized access, in compliance with the platform’s security policies.

Statistical analysis was performed using SurveyMonkey (accessed in June 2022), which provided a descriptive summary of all variables. Data analysis was further carried out using Excel statistical software, and percentage values were reported following tabulation.

## Results

### First section

Responses were received from 104 of the 183 surveyed centers. The participating centers were evenly distributed across all Italian regions, with the highest representation from Northern Italy (39%), followed by Southern regions and islands (35%) and Central Italy (26%). The respondents had a median age of 57 years (range 34–74), with 64 participants (62%) being male and 40 (38%) female.

The majority of the responding physicians were from General Hospitals (64%), followed by those from Private Contracted/Private Institutes (22%) and University Hospitals (14%). Regarding work schedules, 73% of Ros reported that their centers generally operate 5 days/week, from Monday to Friday.

The median number of linear accelerators (LINACs) per center was 2 (range 1–6), with 69% of machines installed after 2010. Notably, 16% (*N* = 17) of centers reported operating with only one LINAC, while 45% (*N* = 47) of respondents indicated that their centers utilized dosimetrically equivalent LINACs. Further details on respondents and their respective centers are presented in Table 1.

### Second section

ROT interruptions were perceived as a critical management issue by 96% of respondents, particularly in the context of curative (51%) or adjuvant treatments (29%). Among different cancer types, head and neck (23%), gynecological (20%), lung (18%), and rectal cancers (15%) were considered the most sensitive to treatment interruptions.

Regarding the duration of interruptions, 42% of respondents defined a treatment break of more than 5 days as critical. In addition, 63% of participants believed that an interruption could have a negative impact on treatment outcomes regardless of when it occurred (whether at the start, midway, or end of the treatment course).

Only 29% of Italian Ros reported following any formal guidelines for RT interruptions. Among those who did, the most commonly referenced sources were the Royal College of Radiographers (RCR) guidelines or relevant literature articles. Further details are provided in Table 2.

### Third section

Approximately 24% of ROs reported adhering to codified procedures for managing RT interruptions at their centers, while 84% indicated that patients were regularly monitored for treatment breaks.

**Fig. 1:**
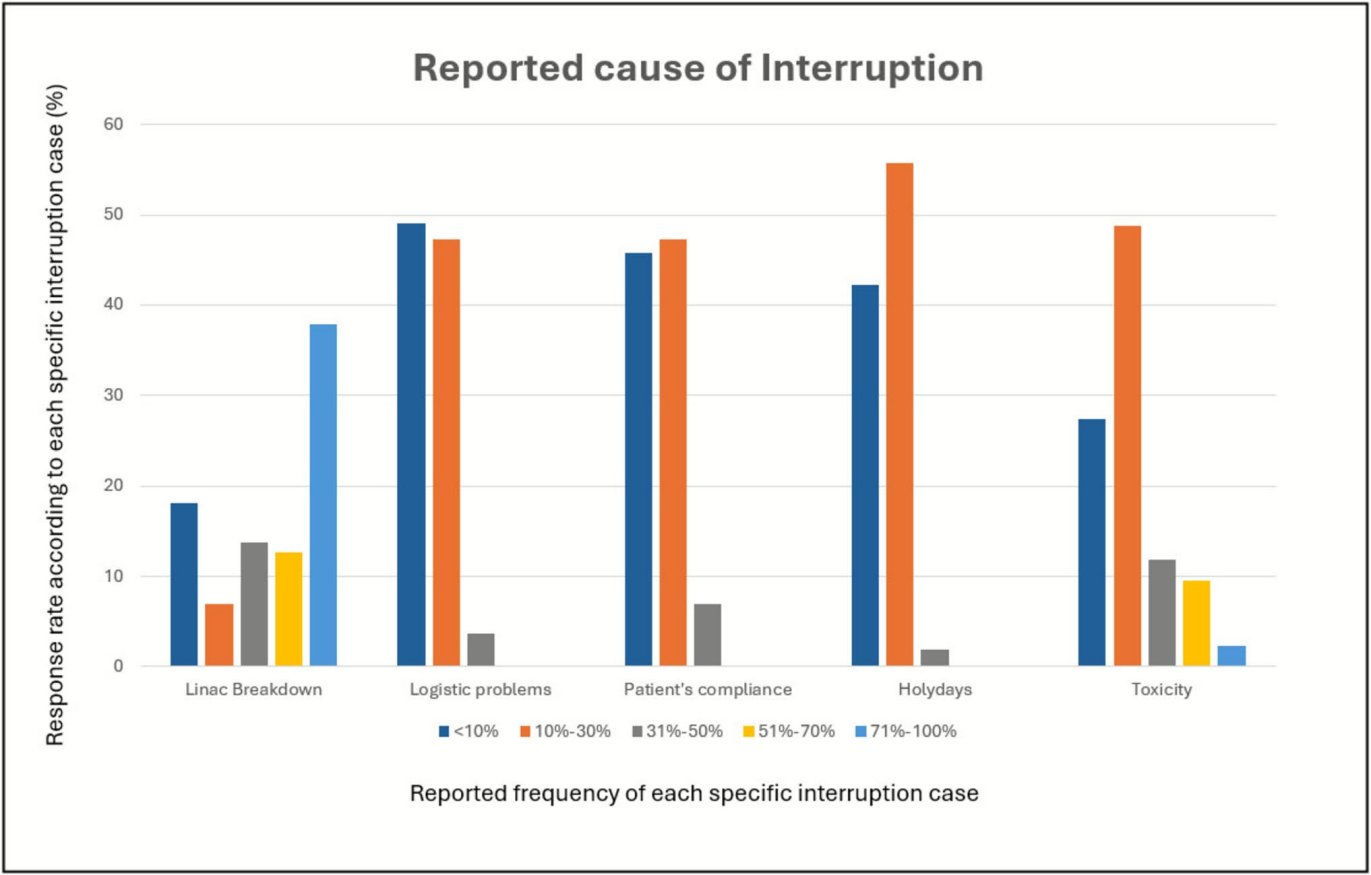
Response interruption percentages according to the main causes of radiation therapy breaks

The most frequently cited causes of RT interruptions were LINAC breakdowns (22%), treatment-related toxicity (22%), and patient compliance issues (22%). Figure 1 illustrates the distribution of interruption causes, highlighting LINAC failures and patient-related toxicities as the most prevalent factors.

#### Impact of COVID-19 on RT interruption management

The survey also assessed how ROT interruptions were managed during the COVID-19 pandemic, both before and after the introduction of vaccines. Before vaccine availability: (i) only 14% of ROs continued treatment for COVID-19-positive patients; (ii) 48% suspended treatment, irrespective of symptoms; (iii) 28% suspended treatment only for symptomatic patients. After vaccines became available, the percentage of Ros continuing treatment increased to 20% and those suspending treatment only for symptomatic patients rose to 43%.

#### Management of LINAC breakdowns

For centers operating with a single LINAC (*N* = 17 centers), only 24% of ROs had agreements with other centers to ensure patient treatment continuity. In this setting, 59% of respondents identified the Radiographers’ Coordinator and/or the Expert in Medical Physics as the main coordinating figures for management of LINAC breakdowns.

All ROs confirmed that their centers had a preventive maintenance contract with LINAC manufacturers. The maintenance was primarily conducted by the equipment manufacturer (97%) and scheduled on any day of the week (98%).

For centers with multiple LINACs (*N* = 85 centers), 82% of ROs reported switching patient treatments to an alternative LINAC in case of a breakdown. However, among these, 75% (*N* = 64/85) applied this strategy selectively, primarily in cases involving concomitant systemic therapy (28%), primary tumor treatments (26%), or exclusive/adjuvant RT (25%). Moreover, although 72 out of 85 centers reported following specific procedures for these cases, only 18 Ros had a written, formalized protocol in place.

#### Compensation strategies for RT interruptions

When switching to another LINAC was not possible, 72% of respondents considered replanning a backup strategy, but only 8% had a predefined rescue RT plan at the start of treatment.

Regarding dose recovery strategies: (i) 28% of Ros always compensated for missed doses following an RT interruption; (ii) 59% reported doing so occasionally. Furthermore, the most frequently used dose recovery strategy was increasing the total dose while maintaining the same fractionation (48%), followed by extending treatment to Saturdays (20%).

For centers implementing fractionation modifications, 76% of ROs informed patients of the change, while 20% required patients to sign a new informed consent. More detailed data are presented in Table 3.

## Discussion

The present study highlights that the management of unscheduled ROT interruptions remains an unmet need across Italy, with significant variability in approaches among RO centers. The lack of standardized protocols has led to inconsistencies in how interruptions are defined, managed, and compensated, potentially impacting patient outcomes.

The responding ROs represented a diverse range of public, private, and contracted facilities across the country, with a median of two LINACs per facility. The majority of ROs identified head and neck (23%), cervical (20%), lung (18%), and rectal (15%) cancers as the most critical settings for RT interruptions, particularly in curative and adjuvant treatments. This finding aligns with prior reports from the 1980s and 1990s [22–29], when less-advanced treatment technologies were available, as well as with more recent studies, such as de la Vega et al. [2], which identified radical/adjuvant RT in head and neck, gynecological, and lung cancers as the most commonly interrupted treatments. This trend is likely explained by the high-dose requirements of radical treatments, where treatment continuity is essential for achieving curative outcomes. Notably, evidence suggests that for cancers such as head and neck and cervical cancer, even a loss of 1 Gy can significantly reduce local control, further underscoring the clinical implications of interruptions [2–4].

### Variability in defining critical ROT interruption durations and timing

One of the most striking findings of our study is the heterogeneity in how ROs define a critical interruption and whether the timing of the interruption influences treatment outcomes. The majority of Italian ROs considered an interruption of 5 or more days to be clinically significant, while 63% believed that any break, regardless of when it occurs, negatively impacts outcomes.

However, the literature suggests a more nuanced picture. The optimal threshold for defining a critical ROT interruption remains debated, with most available data originating from pre-2000 studies that presented significant facility-related biases. Despite this, research has shown that a treatment prolongation of just 1 week can lead to a relative loss of local control ranging from 3 to 25% [30–32]. More specifically, for head and neck, gynecological, and lung cancers, an unscheduled 1-day interruption, if left uncompensated, may result in an absolute local control reduction of 1.0–1.4% [23–34].

Another area of debate in the literature concerns whether the timing of an interruption matters. Some retrospective studies suggest that the phase of treatment during which the interruption occurs (early, midway, or late) does not significantly impact outcomes [23, 35–37]. However, these findings were based on heterogeneous patient populations treated with outdated technology, making them susceptible to considerable biases. Conversely, it is well established that accelerated repopulation, which begins in some tumors after 28 days of ROT, can significantly alter the K-factor (a measure of radiation dose loss due to tumor repopulation) [20]. This suggests that interruptions occurring beyond this threshold may be particularly detrimental, although further research is needed to refine this concept.

Interestingly, only 29% of Italian ROs reported following specific guidelines (e.g., RCR guidelines), indicating that many clinicians rely on personal experience rather than standardized protocols. Given the well-documented impact of ROT breaks, there is an urgent need to establish clear, evidence-based recommendations to guide clinicians in managing and compensating for treatment interruptions.

### Management of ROT interruptions and feasibility of compensation strategies

Although 84% of respondents reported regularly monitoring ROT interruptions, only 24% had formalized protocols in place. This finding is particularly concerning, as structured monitoring programs with codified procedures have been shown to reduce treatment delays and optimize dose compensation, as demonstrated in studies by de la Vega et al. [2] and Pozo et al. [38].

The primary causes of ROT interruptions identified in our study—LINAC breakdowns (22%), toxicity (22%), and patient compliance issues (22%)—are consistent with findings reported in the literature. However, national holidays have been described as a major cause of interruptions in other settings [2, 38, 39], highlighting cultural differences in ROT scheduling.

The COVID-19 pandemic also influenced the management of ROT interruptions. Before vaccine availability, only 13% of ROs continued treatment for COVID-positive patients, whereas after vaccine introduction, 20% of ROs continued treatment and 43% suspended it only for symptomatic patients. This shift reflects an evolving, risk-adapted approach, similar to findings from a previous AIRO survey conducted in 2020 [21].

Significant variability was also observed in how treatment interruptions were compensated. In accordance with the RCR guidelines [40], the most frequently recovered treatments were those with radical intent. When a LINAC breakdown occurred, the preferred strategy was to switch patients to another LINAC, whereas for other causes of interruptions, the most common recovery method was to increase the total dose without altering fractionation [40].

However, other strategies—such as Saturday treatment sessions or twice-daily fractionation—were less commonly employed, likely due to logistical barriers such as: (i) staff availability; (ii) equipment scheduling and facility operating hours; (iii) patient transportation challenges; (iv) institutional policies and financial constraints; and (v) coordination with multidisciplinary teams.

Given these limitations, alternative strategies could be explored, including: (i) proactive hypofractionation regimens to mitigate the effects of interruptions; (ii) regional RT networks to allow patient transfers during LINAC failures; and (iii) telemedicine-based compliance monitoring to address patient-related interruptions at an early stage.

### Comparison with international approaches and future directions

Internationally, RCR guidelines [40] provide structured recommendations. Italy lacks a national protocol, underscoring the need for AIRO-led initiatives to develop evidence-based guidelines.

Future research should focus on: (i) multi-institutional studies assessing the impact of different ROT interruption durations; (ii) AI-driven predictive models to stratify patients based on risk and guide compensation strategies; (iii) feasibility studies on alternative compensation approaches (e.g., weekend treatments, adaptive replanning).

### Strengths and limitations

This study provides valuable insights into the current landscape of ROT interruption management across Italy, offering one of the most comprehensive national assessments on this topic to date. A key strength lies in the large and geographically diverse sample, which includes public, private, and university-affiliated centers, making the findings broadly representative of Italian ROT practice. In addition, the study captures real-world clinical perspectives, highlighting both common challenges and variability in clinical decision-making.

However, several limitations should be acknowledged. First, as a survey-based study, responses may be influenced by recall bias or subjective interpretation of ROT interruptions. Second, centers that did not participate may have different approaches, potentially limiting the generalizability of the findings. Lastly, the study does not include longitudinal clinical outcome data, which would be necessary to quantify the actual impact of different interruption management strategies on patient prognosis.

Future studies incorporating prospective clinical data and comparative analyses of compensation strategies could further refine best practices and support the development of national guidelines.

## Conclusion

This study highlights unplanned ROT interruptions as a significant and unresolved issue in Italian RT practice. The absence of national guidelines has led to inconsistent management approaches, potentially compromising treatment outcomes.

Developing standardized national protocols could be a crucial step in ensuring equitable, high-quality ROT care across Italy. By fostering collaboration among centers, promoting structured monitoring, and integrating modern radiobiological insights, the Italian ROT community can move toward a more effective and uniform approach to managing ROT interruptions.

## Supplementary Information

Below is the link to the electronic supplementary material.

Supplementary file1 (DOCX 19 KB)

## Data Availability

It is not applicable because all the collected data are reported in this paper.
