# Supplementary material for: Managing unplanned radiotherapy interruptions in Italy: results from an AIRO survey
Source: Clin Transl Oncol. 2026 Feb 1;28(7):2911–20. doi: 10.1007/s12094-025-04211-6 (PMC13282210; doi:10.1007/s12094-025-04211-6)
Supplement: Supplementary file 1 — Supplementary file1 (DOCX 19 KB) [file 12094_2025_4211_MOESM1_ESM.docx]

Radiotherapy Treatment Interruptions Survey.

(addressed to all Italian radiotherapy center directors/managers)

1. Genre

a. Woman

b. Male

2. Age: _______________

3. Membership structure:

a. Public hospital

b. University Hospital

c. Private contracted

d. Private

4. Region: _________________

5. No. of linear accelerators present at your center: ___________

6. What is the year of installation of the equipped linear accelerators in the center where you work?

a. ____

b. ____

c. ____

d. ____

e. ____

7. In the center where you work, for concomitant therapy management and supportive therapy (multiple answers possible):

a. The radiotherapy department has regular inpatient beds

b. The radiotherapy department has DH beds

c. The Radiotherapy department directly manages supportive therapy and am-bulatory systemic therapies

d. Supportive therapy and systemic therapies are managed by other specialists in their departments

e. ________________________________________________________________

8. In the center where you work the activity is carried out:

a. 5 days/week

b. 6 days/week

c. 5 days/week with possible recovery on Saturdays

9. In the center where you work, patients start radiation treatment:

a. On Mondays

b. On Tuesdays

c. Any day of the week

d. ___________________________________________

10. Do you consider interruptions in radiation treatment an issue to be managed?

a. Yes

b. No

11. If you answered yes to the previous question, in what clinical situations (multiple answers possible)?

a. For radical treatments

b. For palliative treatments

c. For adjuvant treatments

12. If you answered yes to question #10, in which oncological conditions (multiple answers possible)?

a. Breast tumors

b. Tumors of the prostate

c. Tumors of the uterine cervix

d. Tumors of the rectum

e. Tumors of the H&N district

f. Tumors of the encephalon

g. Tumors of the lung

h. _________________________________________________________

13. How many days of treatment interruption do you think would have a negative impact on the purpose of treatment:

a. 2 days

b. 3 days

c. 5 days

d. > 5 days

14. Do you think that stopping the treatment may have a negative impact on the purpose of the treatment:

a. At any stage of the processing

b. At the initial stage

c. In the middle of the treatment

d. In the final stage of treatment

15. Do you follow literature references/Guidelines on the management of treatment interruptions and dose recovery?

a. No

b. Yes: (Specify) ______________________________________________________________

16. In the center where you work, are there written procedures on the management of treatment discontinuation?

a. Yes

b. No

17. Are patient treatment interruptions monitored in the center where you work?

a. Yes

b. No

18. If yes, what are the most frequent causes of treatment discontinuation in the center where you work (give percentages for each of the items):

a. Equipment failure: _______ %.

b. Logistical problems _____________ %.

c. Poor patient compliance _____________ %

d. Holidays _____________ %

e. Toxicity of treatments ___________ %

f. Other: ___________________________ %

19. During the Covid-19 pandemic period, also based on your hospital's health guidelines before the advent of vaccines:

a. Did you discontinue treatment of all Covid-19 positive patients until negative

b. You stopped treatment of only symptomatic Covid-19 positive patients

c. You have not stopped treatment of any Covid-19 patients

d. ____________________________________________________________

20. After the advent of vaccines:

a. You stopped treatment of all Covid-19 positive patients until they became negative

b. You stopped treatment of only symptomatic Covid-19 positive patients

c. You have not stopped treatment of any Covid-19 patients

d. ______________________________________________________________

21. If only one linear accelerator is present, are there formalized agreements with other centers to treat patients during downtime

a. Yes

b. No

22. Are the linear accelerators equipped at the center where you work 'dosimetrically twin'?

a. Yes

b. No

23. In case of downtime do you treat patients by moving them to another linear accelerator?

a. Yes

b. No

24. In case of previous yes answer, move to another linear accelerator:

a. All patients

b. Only some

25. Follow precise criteria in making the selection of patients to move to another accelerator:

a. Yes, formalized in procedures

b. Yes, not formalized

c. No

26. If the previous answer is yes, indicate the criteria you follow (multiple answers are possible):

a. Primary tumor

b. Exclusive/adjuvant treatment

c. Symptomatic/non-symptomatic treatment

d. Concomitant systemic treatment

e. Other (please indicate)

_____________________________________________________________________

_____________________________________________________________________

27. Before moving the patient to a second Linac recalculate on the TPS the Treatment Plan?

a. Yes

b. No

28. Calculate the treatment plan for 2 different linear accelerators before the start of treatment

a. Yes

b. No

29. In the center where you work, is recovery of the dose not administered as a result of the interruption carried out?

a. Yes, always

b. Yes, in sporadic cases

c. No, never

d. No, I only recover the missed session(s) by extending the total treatment time

30. If the previous answer is yes, how is the unadministered dose recovered (multiple answers are pos-sible):

a. By retrieving the dose(s) on the following Saturday(s)

b. By making two daily applications on one or more days

c. Using an accelerated regimen (hyperfractionated or not) in the remaining part of the treatment, to keep the overall treatment duration constant

d. Changing (increasing) the total dose, to recover the lost dose, without changing the fra-ctionation

e. By modifying (increasing) the total dose, to recover the dose lost, by modifying the fractionation (e.g., hyperfractionation)

31. In case a different daily fractionation is used to recover the lost dose, do you inform the patient?

a. Yes, without submitting a new consent because the possibility of fractionation change is described as possible in the Informed Consent

b. Yes, by submitting a new Informed Consent to him or her

c. No

32. In the center where you work who handles the equipment failure:

a. Clinical engineering

b. The technical department

c. The coordinator of medical radiology health technicians

d. Altro_______________________

33. Do the linear accelerators supplied to the center at which you work have a regular preventive maintenance contract?

a. Yes

b. No

34. If you answered yes to the previous question, preventive maintenance of the equipment is carried out:

a. By the manufacturer of the equipment

b. By the technical service department

c. By an external service
